# Sleeping Habits

**Published:** 1869-02

**Authors:** 


### SLEEPING HABITS.

To be able to lie down at night and fall to sleep within ten minutes, and to know no dream or waking until the morning comes, and then to bound out of bed full of health, freshness and good humor, is a blessing well worthy the warmest outgushings of a thankful heart towards Him who giveth us all things richly to enjoy.

Some of the ways of attaining such a priceless boon we here name:

Take dinner at the good old-fashioned hour of mid-day; eat nothing afterwards, except at supper, when a piece of cold bread and butter with a single cup of weak tea or half a glass of pure water is enough for anybody under ordinary circumstances. If dinner is taken in the middle of the afternoon, do not eat an atom of anything until next morning. Another plan is, avoid sleeping in the day-time, and retire habitually at a regular hour.

In order to make a desirable result more certain, remember practically the following facts:

We need, ordinarily, seven hours of sleep in summer and eight in winter.

We breathe in sleep about fifteen times every minute.

Each inhalation of pure air is returned loaded with poison; a hundred and fifty grains of it is added to the atmosphere of a bedroom every hour, or twelve hundred grains during a night.

Unless that poison-laden atmosphere is diluted or removed by a constant current of air passing through the room, the blood soon becomes impure, then circulates sluggishly, accumulating and pressing on the brain, giving rise to frightful dreams. If the room is small and tight, the spectral nightmare, the fearful groan, the terrible shriek, are the result, and in aggravated cases, with the addition of a hearty late meal, there is not strength to give the moan, to raise the shriek, and thus arouse the system; there is no power to move, the man feels a crushing danger coming upon him, he can 't get out of the way. "Found dead in his bed!" that is the morning's verdict.

